# The Value of Sleep for Women

**Published:** 1891-04

**Authors:** 


### THE VALUE OF SLEEP FOR WOMEN.

Our American girls lay too little stress upon the value of sleep as the best and most wonderful tonic to the human system. It is no uncommon thing for them to be up until midnight or later, and yet arise in time to breakfast with the family at the usual hour, eight o'clock in the morning. The parents are somewhat to blame in this matter. Many of them have still the old fashioned idea that lying in bed in the morning is a form of idleness that should not be indulged in; and fathers, particularly, are most apt to feel that their daughters are inattentive if they are not on hand to brighten the breakfast hour and give them a good morning kiss. And it is a hardship, but a necessary one, if we would have our daughters retain their health and beauty.

An unusually handsome St. Louis woman, says the *Post-Despatch*, of that city, who has at the age of almost fifty years the fine well rounded figure and the elastic step and carriage of a girl, the delicate rose-hued skin, and the brilliancy of youth in her eyes, says that she has made it a rule to retire at nine o'clock, except on very rare occasions ; and then she takes a nap in the afternoon to prevent the ill effects of the late hours which are to follow.

Our American women of all classes need, more than any other people in the world, the rest and refreshment which only sleep can give to overwrought nerves and overworked systems, for nowhere else do the women live under so much physical and mental strain. To some natures sleep does not come easily. In that event, some light exercise should be taken nightly before retiring, directing the blood thereby into proper channels, when sleep will come readily as to a tired child. What women need most is a knowledge of self, and an intelligent understanding of nature's laws, not a parcel of nostrums of which they know nothing, and which may be hurtful in the extreme.—*Boston Jour. Health.*

